# T cell-targeting nanoparticles focus delivery of immunotherapy to improve antitumor immunity

**DOI:** 10.1038/s41467-017-01830-8

**Published:** 2017-11-23

**Authors:** Daniela Schmid, Chun Gwon Park, Christina A. Hartl, Nikita Subedi, Adam N. Cartwright, Regina Bou Puerto, Yiran Zheng, James Maiarana, Gordon J. Freeman, Kai W. Wucherpfennig, Darrell J. Irvine, Michael S. Goldberg

**Affiliations:** 1 Department of Cancer Immunology & Virology, Dana-Farber Cancer Institute, Boston, MA 02215 USA; 2 Department of Microbiology & Immunobiology, Harvard Medical School, Boston, MA 02215 USA; 3 Department of Biological Engineering, MIT, Cambridge, MA 02139 USA; 4 Koch Institute for Integrative Cancer Research, Cambridge, MA 02139 USA; 5 Howard Hughes Medical Institute, Chevy Chase, MD 20815 USA

## Abstract

Targeted delivery of compounds to particular cell subsets can enhance therapeutic index by concentrating their action on the cells of interest. Because attempts to target tumors directly have yielded limited benefit, we instead target endogenous immune cell subsets in the circulation that can migrate actively into tumors. We describe antibody-targeted nanoparticles that bind to CD8^+^ T cells in the blood, lymphoid tissues, and tumors of mice. PD-1^+^ T cells are successfully targeted in the circulation and tumor. The delivery of an inhibitor of TGFβ signaling to PD-1-expressing cells extends the survival of tumor-bearing mice, whereas free drugs have no effect at such doses. This modular platform also enables PD-1-targeted delivery of a TLR7/8 agonist to the tumor microenvironment, increasing the proportion of tumor-infiltrating CD8^+^ T cells and sensitizing tumors to subsequent anti-PD-1. Targeted delivery of immunotherapy to defined subsets of endogenous leukocytes may be superior to administration of free drugs.

## Abstract (web summary)

Targeted delivery of immunomodulatory compounds to defined subsets of endogenous immune cells may improve the efficacy of combination immunotherapies. Here, the authors use PD-1-targeting nanoparticles containing a TGFβ inhibitor or a TLR7/8 agonist to deliver these payloads to T cells or via T cells to the tumor microenvironment, respectively, leading to anti-tumor efficacy in vivo.

## Introduction

Clinical data have shown that stimulation of a patient’s dormant immune system can impart durable benefit against cancer^1^. The proportion of patients who respond to cancer immunotherapy, however, remains modest (<20%). Moreover, systemic immune stimulation is often associated with autoimmune-type pathologies, such as colitis and pneumonitis^2, 3^, as the doses required to break immune tolerance to the tumor can invoke undesired host-vs.-host effects. The ability to concentrate the action of immunostimulatory drugs on tumor-reactive effector cells would improve both efficacy and safety, preventing stimulation of both immunosuppressive cells and non-tumor-reactive effector cells. To this end, we have developed nanoparticles that can target the delivery of immunotherapies to specific subsets of endogenous immune cells. Following intravenous administration, these particles bind to T cells in the circulation, which actively migrate to solid tumors and can carry the particles into the harsh, immunosuppressive tumor microenvironment.

TGFβ is a major mediator of immunosuppression^4^, but systemic administration of TGFβR1 inhibitors can be toxic owing to the importance of this signaling pathway in disparate cellular contexts^5^. The role of TGFβ signaling specifically in T cells was recently demonstrated using mice expressing a dominant-negative form of TGFβRII, which has a truncated intracellular kinase domain that outcompetes the endogenous receptor for heterodimerization with TGFβRI. This cell type-restricted signal inhibition reduced medulloblastoma progression by limiting the activity of regulatory T cells (Tregs) as well as promoting the expansion and activation of CD8^+^ T cells^6^. We hypothesized that release of SD-208, a TGFβR1 inhibitor, in an autocrine-like manner from PLGA nanoparticles targeted to T cells would restore effector T cell function and thereby enable robust killing of cancer cells. Moreover, we hypothesized that paracrine-like release of SD-208 within the tumor microenvironment could rescue the function of other suppressed immune cells. Notably, the antibody fragments used to target the nanoparticles to the cells of interest can also be used to impart immune checkpoint blockade, thereby further augmenting the functionality of exhausted T cells, such as those expressing PD-1.

The particles described herein have been designed to increase the proportion of patients who respond to immunotherapy and to minimize the side effects that they experience. These particles have strong potential for clinical translation as they are prepared from the FDA-approved polymers poly(lactic-co-glycolic acid) (PLGA) and polyethylene glycol (PEG). PLGA/PEG-based nanoparticles have previously been used to target the delivery of cytotoxic chemotherapy^7^ or molecular targeted therapy^8^ to cancer cells based on binding to receptors expressed on their surface.

Unfortunately, directly targeting receptors on the surface of cancer cells does not seem to work as well as had been hoped, as targeted and untargeted particles exhibit similar biodistribution and tumor localization patterns^9^. Most nanoparticles rely on passive accumulation into tumors, and their efficacy has been most pronounced in preclinical models of solid tumors that harbor leaky vasculature^10^, which may not reflect tumors that grow over the course of years rather than days. In contrast, immune cells traffic actively down chemokine gradients to sites of inflammation, such as tumors. Indeed, leveraging T cells as vectors greatly enhances the quantity of drug that can be delivered to tumors, achieving levels in the tumor that are orders of magnitude greater than that which can be delivered by nanoparticles alone^11^. Furthermore, most approaches to date have focused on the delivery of cytotoxic agents, which must kill the vast majority of the target cells in order to be effective. Much lower concentrations of immunomodulatory drugs are required, as such compounds can stimulate an amplifying response.

The conjugation of drug-containing liposomes to the surface of T cells prior to adoptive cell transfer dramatically improves the potency of the administered cells^12, 13^. The liposomes, however, become diluted as the cells proliferate. It has also been shown that adoptively transferred T cells can be effectively targeted in vivo by antibody-functionalized or cytokine-functionalized liposomes, enabling repeated expansion of the transferred cells^14^. Here, we sought to demonstrate that targeting of endogenous immune cells could be achieved in the absence of the cumbersome and costly procedures associated with adoptive cell transfer. We further aimed to demonstrate that we could deliver small molecule immunomodulators in a targeted manner via these nanoparticles.

We hypothesized that delivery of immunomodulatory compounds via T cell-targeting nanoparticles would augment T cell function better than systemic administration of free drug. To this end, we show that particles can be targeted to particular endogenous T cell subsets in blood, secondary lymphoid organs, and tumors. Importantly, the particles can be targeted to surface receptors in a modular manner, as we confirm targeting of lineage markers (e.g., CD8) as well as functional markers (e.g., PD-1). We show specific binding in vitro and in vivo. This modularity extends to the entrapped payload, as the particles can be loaded with a variety of small molecule drugs, which are released from the particles in a sustained manner. Targeted delivery of an inhibitor of TGFβ signaling to PD-1-expressing T cells delays tumor growth and extends the survival of mice harboring colorectal tumors relative to administration of free drug. Importantly, targeted delivery of a TLR7/8 agonist to PD-1-expressing T cells can recruit lymphocytes to non-inflamed tumors, providing a novel off-the-shelf approach to improving the percentage of patients who respond to cancer immunotherapy.

## Results

### Generation of nanoparticles targeting CD8^+^ T cells

**Fig. 1:**
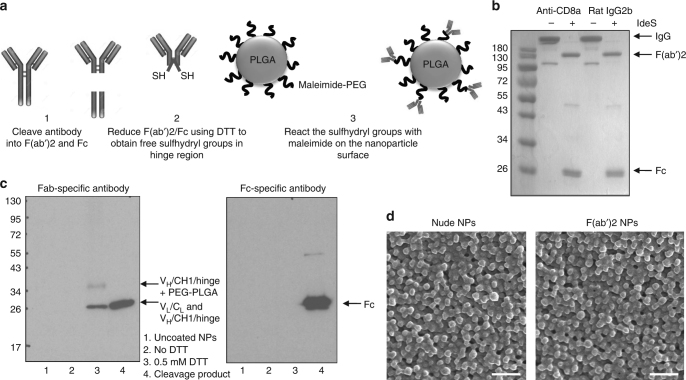
Optimization of F(ab’)2 conjugation to polymeric nanoparticles. **a** Scheme of antibody fragment conjugation to the surface of pre-formulated maleimide-functionalized PEG-PLGA polymeric nanoparticles (NPs). **b** A non-reducing SDS–PAGE gel stained with Coomassie Brilliant Blue is shown following IdeS-mediated cleavage of anti-CD8a and rat IgG2b isotype control antibodies. **c** A Western blot following reducing SDS–PAGE of CD8a-targeting nanoparticles developed with Fab-specific (left panel) or Fc-specific antibodies (right panel); lane 1: uncoated nanoparticles, lane 2: nanoparticles without antibody reduction before conjugation, lane 3: anti-CD8a nanoparticles with the antibody reduced using 0.5 mM DTT before conjugation, lane 4: anti-CD8a F(ab’)2 and Fc cleavage product as a positive control. **d** Scanning electron microscopy images of blank NP formulations before and after F(ab’)2 conjugation; scale bar = 500 nm

In this study, we aimed to develop nanoparticles that bind specifically to particular T cell subsets in order to concentrate immunomodulatory drugs to these cells or to leverage the cells as carriers of the payload into the tumor microenvironment. To establish proof-of-concept, we began our studies with particles that could target CD8^+^ T cells, as there are numerous reagents available to isolate and identify this population and due to the high receptor density of CD8 on T cells. CD8^+^ T cell-specific nanoparticles were generated by conjugating anti-CD8a F(ab’)_2_ fragments to the particle surface. These antibody fragments were produced by IdeS-mediated cleavage of full-length IgG molecules. High target specificity and avidity were thus achieved in the absence of potential interactions with Fc receptors expressed by phagocytic cells, which are a major means of nanoparticle clearance^15^. Following the sequence-specific cleavage of the antibody below its hinge region, the disulfide bonds were reduced, and the resulting sulfhydryl groups were reacted with maleimide-functionalized PEGylated PLGA nanoparticles (scheme shown in Fig. 1a).

IdeS cleaved rat IgG2b antibodies (anti-CD8a and isotype control) with greater than 95% efficiency (Fig. 1b), and Western blot analysis confirmed that reduction of disulfide bonds (with 0.5 mM dithiothreitol) was required for conjugation of (Fab’)2 fragments (Fig. 1c, lanes 2 and 3 of left panel). Moreover, this analysis showed that the Fc portion that remained present in the reaction mixture as cleavage product was not conjugated to the nanoparticle surface (Fig. 1c, lane 3 of right panel compared to positive control in lane 4). The addition of F(ab’)2 did not lead to a significant increase in nanoparticle size (269 ± 8 nm for Iso NPs and 273 ± 8 nm for anti-CD8 NPs, *n* = 8 ± s.d.) relative to uncoated nanoparticles (267 ± 8 nm, *n* = 9 ± s.d.), as determined by dynamic light scattering. A homogeneous distribution with similar size and morphology of nude and F(ab’)2-conjugated nanoparticles was also confirmed by scanning electron microscopy (SEM) (Fig. 1d). The zeta potentials of nanoparticle formulations were determined before and after F(ab’)2 conjugation with all drugs and dyes that were encapsulated for these studies (Supplementary Table 1). Although the conjugation of F(ab’)2 does not affect the zeta potential markedly, the payload can alter the charge measured.

### Binding to CD8^+^ T cells is specific in vitro and in vivo

**Fig. 2:**
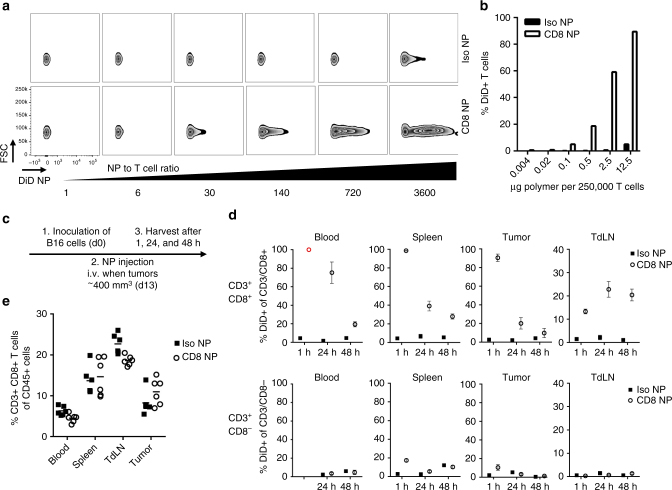
CD8a-targeting nanoparticles bind to T cell in vitro and in vivo. **a** CD8a-targeting nanoparticles (loaded with DiD), but not isotype control nanoparticles, bind to the surface of CD8^+^ T cells isolated from the spleen within 30 min of incubation, as assessed by flow cytometry. **b** Quantification of DiD-positive T cells; data representative for more than 4 experiments. **c** Timeline of in vivo binding experiment. **d** Quantification of DiD-positive, CD3^+^/CD8^+^ and CD3^+^/CD8^−^ T cells 1, 24, and 48 h after the nanoparticles were injected intravenously; *n* = 3 for 1 and 48 h, *n* = 6 for 24 h, mean ± s.e.m.; (red open circle) Anti-CD8a antibody for flow cytometry staining could not bind due to steric hindrance with CD8a-targeting nanoparticles. **e** Quantification of CD3/CD8+ T cells in blood, spleen, tumor-draining lymph node (TdLN), and tumor 24 h after the nanoparticles were injected intravenously; *n* = 6

Having developed a formulation protocol, we aimed to confirm that the particles could bind specifically to the target cells of interest. The CD8a-targeting nanoparticles bound to CD8^**+**^ T cells, enriched from murine spleens, in a dose-dependent manner (Fig. 2a). At nanoparticle to T cell ratios greater than 3600:1, up to 90% of the T cell population were bound by CD8a-targeting nanoparticles, with very little non-specific binding observed by isotype control nanoparticles (Iso NPs) (Fig. 2b). Ovalbumin-specific OT-I CD8^+^ T cells retained their ability to proliferate in the presence of ovalbumin-expressing B16 melanoma cells when nanoparticles were bound to their surface (Supplementary Fig. 1a). Although the anti-CD8a nanoparticles bind to the surface of CD8^+^ T cells, only a small percentage (~20%) of these nanoparticles are internalized by the cells (Supplementary Fig. 1b).

Next, we confirmed that an endogenous immune cell subset could be targeted in vivo. Nanoparticle binding was confirmed in a subcutaneous model of B16 melanoma. Mice with established tumors (~400 mm^3^) were injected intravenously with CD8a-targeting nanoparticles, and immune cells were recovered from the circulation, spleen, tumor, and tumor-draining lymph node over a timeframe of 48 h (Fig. 2c, gating strategy shown for 24 h in Supplementary Fig. 2a).

1 h after injection, 90–100% of the CD8^+^ T cells in the blood, spleen, and tumor tissue were bound by DiD-labeled CD8a-targeting nanoparticles, as determined by flow cytometry (Fig. 2d, upper panel). Of note, CD8^+^ T cells isolated from the blood after 1 h could not be stained with free fluorescently labeled anti-CD8a antibody, owing to saturating steric shielding by the nanoparticles of the receptors on circulating T cells at this time point. One quarter (27.2 ± 2.4%) of CD3^+^ T cells stained positively for DiD (Supplementary Fig. 2b), and this number corresponds to the fraction of CD8^+^ T cells detected in the unbound isotype control group (26.6 ± 5.8%). Hence, CD8a receptors on T cells in the blood are completely saturated by the CD8a-targeting nanoparticles after 1 h. The percentage of CD8^+^ T cells recovered from blood, spleen, and tumor that are bound by CD8a-targeting nanoparticles decreases over 24 h but persists for at least 48 h. Flow cytometry similarly confirmed that the targeting to CD8^+^ T cells was specific, as binding of targeted and control nanoparticles to CD3^+^CD8^−^ T cells was minimal at 1, 24, or 48 h post-injection (Fig. 2d, lower panel).

Interestingly, the accumulation of CD8a-targeting nanoparticles in the tumor-draining lymph nodes increases over the timeframe evaluated. It is possible that the nanoparticles accumulate passively in the draining lymph nodes and/or that T cells from the blood and/or tumor traffic there. Of note, unlike free anti-CD8a IgG, which results in target cell depletion owing to its isotype^16^, administration of CD8a-targeting nanoparticles does not induce a significant reduction of CD8^+^ T cells (Fig. 2e). These data confirm that the Fc has been effectively removed during the cleavage and conjugation process.

It was next established that CD8a-targeting nanoparticles did not accumulate in the thymus. CD4^+^, CD8^+^, and double-positive T cells recovered from the thymus were not bound by targeted or control nanoparticles loaded with DiD (Supplementary Fig. 3a). However, binding capacity to thymocytes was preserved *ex vivo*. When thymocytes were isolated and incubated with isotype control nanoparticles, ~80% of the CD45^+^ population was double positive for CD4 and CD8. In contrast, when thymocytes were incubated with anti-CD8a nanoparticles, the double-positive population decreased dramatically to ~20% owing to blockade of CD8 receptors (Supplementary Fig. 3b, left panel). The binding was further confirmed by the significant increase in DiD-positive cells in the CD45^+^ population (right panel).

While nanoparticles are often phagocytosed non-specifically by myeloid cells^17^, the targeting particles described herein exhibit very modest interaction with CD11b^+^ immune cells isolated from the blood and tumor 1 and 24 h after intravenous injection (Supplementary Fig. 3c). At 72 h post-injection of nanoparticles targeting Gr-1 (expressed on myeloid-derived suppressor cells) or untargeted isotype controls, no binding whatsoever was observed among F4/80^+^ cells (macrophages), further supporting the lack of off-target binding and/or uptake (Supplementary Fig. 3d). These data confirm that the F(ab’)2-conjugated targeting nanoparticles described herein, which lack Fc that could be recognized by Fc receptors, show little uptake by myeloid cells. This finding may be rather important, as it has recently been shown that anti-PD-1 antibodies (full IgG with intact Fc) are quickly removed from T cells by tumor-associated macrophages, which thereby reduce the efficacy of the immunotherapy^18^.

### Targeting to functional markers can also be achieved

It has been shown that PD-1 identifies the tumor-reactive repertoire of CD8^+^ T cells that infiltrate human tumors^19^ as well as neoantigen-specific CD8^+^ T cells in the peripheral blood of melanoma patients^20^. We thus sought to target PD-1^+^ cells rather than all CD8^+^ cells. Anti-PD-1 was cleaved using IdeZ (Supplementary Fig. 4a), and the absence of Fc on the nanoparticle surface was again confirmed by Western blotting (Supplementary Fig. 4b).

**Fig. 3:**
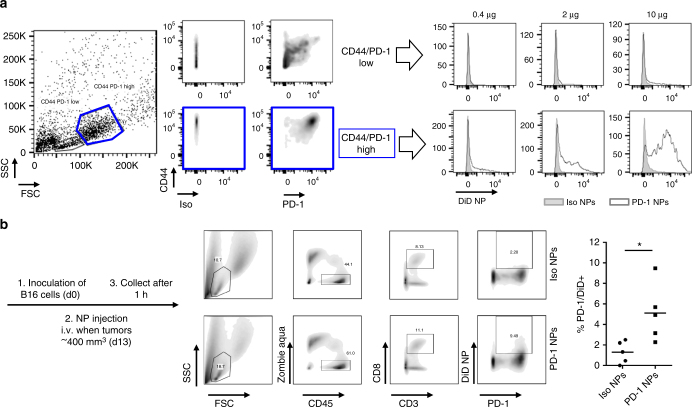
PD-1-targeting nanoparticles bind to T cells in vitro and in vivo. **a** CD8^+^ OT-I T cells were activated with ovalbumin-expressing B16 (ratio 1:10 B16 to T cell) for 48 h and incubated with DiD-loaded, PD-1-targeting nanoparticles for 30 min before detection of DiD by flow cytometry. Mass of polymer indicated is per 250,000 T cells. **b** C57BL/6 mice were inoculated subcutaneously with ovalbumin-expressing B16 melanoma cells. Once tumors reached ~400 mm^3^ in volume, DiD-loaded, PD-1-targeting or isotype control nanoparticles were injected intravenously. 1 h later, tumors were recovered. Flow cytometry was performed (gating shown at left), and the percentage of T cells that were positive for both PD-1 expression and nanoparticle binding was quantified (right panel); *n* = 5 (*p < 0.05, Two-tailed Student’s *t*-test)

Naïve OT-I T cells were activated using ovalbumin-expressing B16 melanoma cells, and cells were gated according to their size and granularity. The smaller and less granular population exhibited lower expression levels of the activation markers CD44 and PD-1, and the binding of PD-1-targeting nanoparticles overlaid with isotype control nanoparticles for these cells (Fig. 3a). In contrast, the bigger and more granular population, which exhibited high expression levels of CD44 and PD-1, exhibited a dose-dependent increase in DiD signal with increasing amounts of anti-PD-1 nanoparticles. Similar results were obtained when the T cells were activated with anti-CD3/CD28 beads (Supplementary Fig. 5).

To assess binding of PD-1-targeting nanoparticles in vivo, mice were inoculated with B16 melanoma cells, and nanoparticles were administered intravenously when the subcutaneous tumors reached a size of ~400 mm^3^. Among immune cells isolated from tumor tissue that was collected 1 h after injection, ~5% of PD-1^+^ T cells were also positive for anti-PD-1 nanoparticles, which was three-fold higher than the baseline observed for control isotype nanoparticles (Fig. 3b). We also found a significant increase (>10-fold) of nanoparticle-positive PD-1^+^ T cells in the blood, though the percentage of PD-1^+^ T cells in the circulation was relatively small at this time point (Supplementary Fig. 6).

### Specific binding to human T cells is observed

**Fig. 4:**
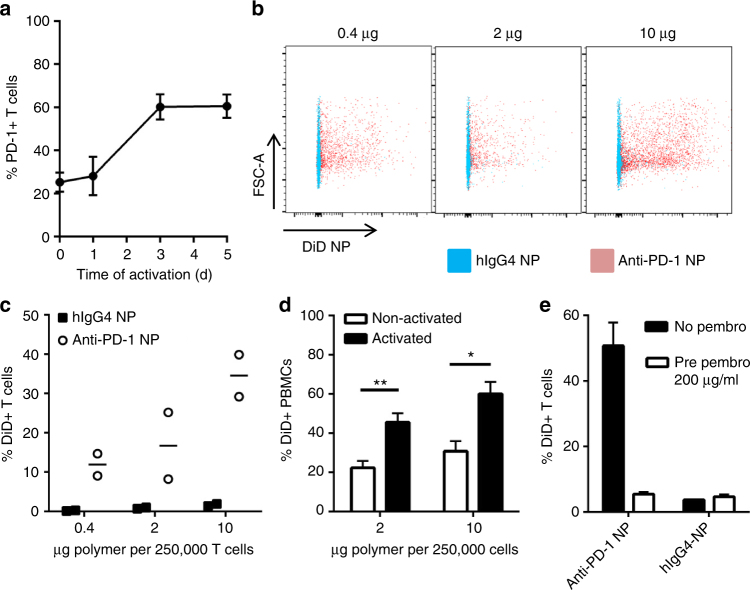
PD-1-targeting nanoparticles bind to activated human T cells. **a** PD-1 expression on human CD3^+^ T cells following activation with anti-CD3/CD28 complex, *n* = 4 independent donors, mean ± s.e.m. **b** Dose-dependent binding of PD-1-targeting nanoparticles to 250,000 activated human T cells. **c** Quantification of T cells that were bound by DiD-loaded, PD-1-targeting nanoparticles; graph shows results of two donors and is representative for at least two independent experiments. **d** Dose-dependent binding of PD-1-targeting nanoparticles to activated and non-activated peripheral blood mononuclear cells (PBMCs), *n* = 4, mean ± s.e.m. **e** Pre-incubation of activated human T cells with free pembrolizumab (pre pembro) for 30 min blocks binding of PD-1-targeting nanoparticles (10 µg per 200,000 T cells; *n* = 3, mean ± s.d.) (*p < 0.05; **p < 0.01, Two-tailed student’s t-test)

To demonstrate the clinical potential of this platform, we confirmed the ability to prepare nanoparticles functionalized with antibody fragments derived from a monoclonal antibody that is used clinically and the ability of such nanoparticles to bind to human T cells expressing the targeted receptor. Pembrolizumab is a fully humanized anti-PD-1 antibody that is approved for the treatment of melanoma^21^, non-small-cell lung cancer^22^, and head and neck cancer^23^. It was successfully cleaved (Supplementary Fig. 7a) and conjugated onto the surface of nanoparticles to assess the potential application of this platform for clinical use. Primary T cells were isolated from healthy human donors, and PD-1 expression was assessed by flow cytometry following activation with anti-CD3/CD28 complexes. PD-1 expression on human T cells increased to 60% by day 3 (Fig. 4a). As no further increase was observed by day five, T cells activated for 3 days were used for further binding studies using fluorescent nanoparticles. Pembrolizumab-coated nanoparticles showed dose-dependent binding to human T cells (Fig. 4b), with up to 40% of the cells being positive for DiD (Fig. 4c). Significantly more nanoparticles bound to activated peripheral blood mononuclear cells (PBMCs) than to non-activated PBMCs (Fig. 4d). This binding was prevented by pre-incubation of the activated T cells with excess competitive free pembrolizumab (Fig. 4e), demonstrating that the binding was specific. Next, we investigated whether PD-1-targeting nanoparticles could be internalized by T cells. Whereas the nanoparticles were detected predominantly on the cell surface for the first 24 h, a reduction of F(ab’)2 signal on the cell surface was observed within the DiD-positive population thereafter (Supplementary Fig. 7b). One interpretation of these data is that up to 50% of the nanoparticles are internalized within 72 h; however, it must be qualified that the DiD may be embedded in the T cell membrane following release from the nanoparticles, so it is also possible that the reduction in F(ab’)2 signal is owing to degradation of the antibody fragments and/or to release of the nanoparticles from the cell surface over time.

### Inhibitor released from particles phenocopies free inhibitor

Having established that the nanoparticles can bind specifically to a defined target in vitro and in vivo, we sought to investigate the impact of targeted delivery of an immunomodulatory small molecule. SD-208 is an inhibitor of TGFβRI kinase^24^ and thereby blocks immunosuppressive pathways induced by TGFβ, which is frequently expressed in tumor tissue^4^. SD-208 is poorly water soluble and is therefore readily entrapped in the hydrophobic core of PEG-PLGA nanoparticles (20 µg/mg polymer). The size and morphology of nanoparticles loaded with SD-208 was confirmed to be very similar to those of empty nanoparticles as well as nanoparticles loaded with DiD or other small molecules (Supplementary Fig. 8a).

**Fig. 5:**
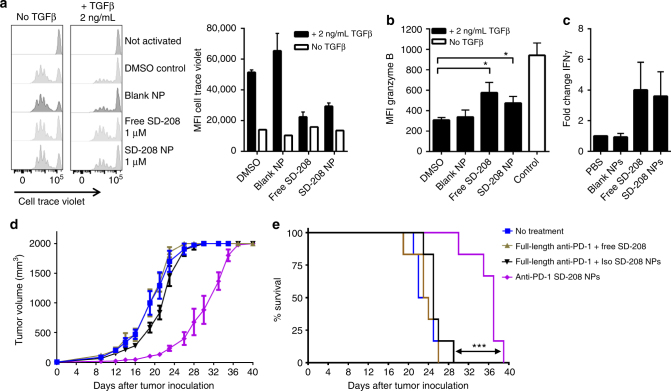
Targeted delivery of a TGFβR1 inhibitor (SD-208) to PD-1-expressing cells delays tumor growth and extends survival. **a** Proliferation of CD8^+^ T cells following activation with anti-CD3/CD28 beads (1:2 bead to T cell ratio) for 72 h in the presence or absence of TGFβ1 (2 ng mL^−1^) and treatment with 1 μM SD-208 as free compound or as nanoparticle formulation; quantification of geometric mean of cell trace violet is provided in the right panel, *n* = 3, mean ± s.d. **b** Intracellular granzyme B expression was assessed by flow cytometry, *n* = 3, mean ± s.d. (*p < 0.05, one-way ANOVA with Tukey’s post hoc test). **c** Fold change of interferon-γ (IFNγ) was measured by ELISA, *n* = 4, mean ± s.e.m. **d**, **e** C57BL/6 were inoculated subcutaneously with MC38 cells. Five days later, nanoparticles or free drugs were administered intravenously every other day up to a total of 10 injections. The dose was 20 μg of anti-PD-1 and 40 μg of SD-208. **d** Tumor volume and **e** animal survival were monitored to assess efficacy, *n* = 6, mean ± s.e.m.; (***p < 0.001, Mantel-Cox test)

The encapsulation efficiency and drug release kinetics of SD-208 were analyzed by its absorbance maximum at 370 nm (Supplementary Fig. 8b). Owing to its limited solubility in aqueous solution, SD-208 is released slowly from the nanoparticles over the course of weeks, as assessed in PBS containing 10% serum (Supplementary Fig. 8c). SD-208 released from nanoparticles conferred similar effects to free SD-208 in cellular assays. Specifically, TGFβ-mediated inhibition of T cell proliferation was reversed in a comparable manner as shown by CFSE dilution (Fig. 5a, left panel) and its mean fluorescence intensity (Fig. 5a, right panel). Moreover, the markers of T cell function granzyme B and interferon γ (IFNγ) were upregulated to a similar extent as free inhibitor in DMSO (Fig. 5b, c). The ability to target the delivery of a TGFβR1 inhibitor to desired cells in vivo represents an intriguing proposition, as systemic inhibition of TGFβR1 awakens dormant disseminated tumor cells, fueling multi-organ metastasis^25^.

### Efficacy is observed only if delivery of payload is targeted

Because anti-tumor immune responses are highly dynamic and coordinated, we transitioned to in vivo studies using the MC38 model of colorectal cancer in order to assess for therapeutic efficacy. MC38 was favored over B16 for in vivo studies because the latter are not greatly affected by anti-PD-1 monotherapy^26^. In contrast, growth of MC38 tumors is delayed by anti-PD-1 monotherapy, albeit at a relatively high dose (300 μg per injection)^27^. SD-208 has similarly been administered at doses more than an order of magnitude greater than what was used in these studies in order to achieve therapeutic benefit, even necessitating daily^28^ or twice daily^29^ oral gavage at such doses (at least 50 mg kg^−1^ per day). We sought to demonstrate that this targeting platform can improve the therapeutic index and achieve efficacy at lower doses, thereby decreasing potential side effects, which remain a challenge in immunotherapy, particularly when multiple agents are being administered. In addition, the biodegradable nanoparticles enable sustained release, improving the pharmacokinetic profile of the small molecule payload.

Mice were inoculated with subcutaneous MC38 tumors and, beginning five days later, were administered anti-PD-1 and SD-208 intravenously at the modest doses of 20 μg anti-PD-1 and 40 μg SD-208, respectively, every other day for up to ten doses. Free anti-PD-1 and SD-208 at these doses had no effect on tumor growth (Fig. 5d) or mouse survival (Fig. 5e). Delayed tumor growth and extended mouse survival were observed if and only if SD-208 was delivered by the PD-1-targeting nanoparticles. In contrast, free anti-PD-1 administered in combination with SD-208 loaded in untargeted nanoparticles had no impact, suggesting that targeted delivery of the small molecule drug was required. In this model, immune evasion ultimately prevailed, as the tumors eventually progressed. To confirm that there was a dose-response, we performed the therapeutic study with twice weekly dosing instead. While the tumor progressed faster, the data confirmed that tumor growth inhibition was inhibited only when the drug was delivered via PD-1-targeting nanoparticles; sundry control formulations—including multivalent anti-PD-1 on blank nanoparticles plus free SD-208—had no effect on tumor progression or mouse survival (Supplementary Fig. 9a, b).

Inspecting for pharmacodynamic markers of drug delivery, we confirmed that the anti-PD-1 F(ab’)2 fragments could induce functional immune checkpoint blockade in addition to defining target specificity. To this end, we observed an increase in the proportion of circulating CD4^+^ T cells, CD8^+^ T cells, and NK cells expressing phosphorylated Zap70, which is indicative of enhanced TCR signaling (that would be inhibited by signaling through PD-1), two days following treatment with PD-1-targeting nanoparticles (Supplementary Fig. 9c). Similarly, we detected an increase in the proportion of NK cells that express IFNγ (Supplementary Fig. 9d). Though we successfully demonstrate the ability to focus the action of a TGFβR1 inhibitor on cells of interest and the benefit thereof, inhibition of TGFβ signaling does not appear to be sufficient to produce curative outcomes in this model. Nonetheless, these data reveal that targeted delivery confers efficacy at more than one logarithm lower dose of both anti-PD-1 and SD-208 than previously reported.

### Targeted delivery of R848 recruits CD8^+^ T cells into tumors

**Fig. 6:**
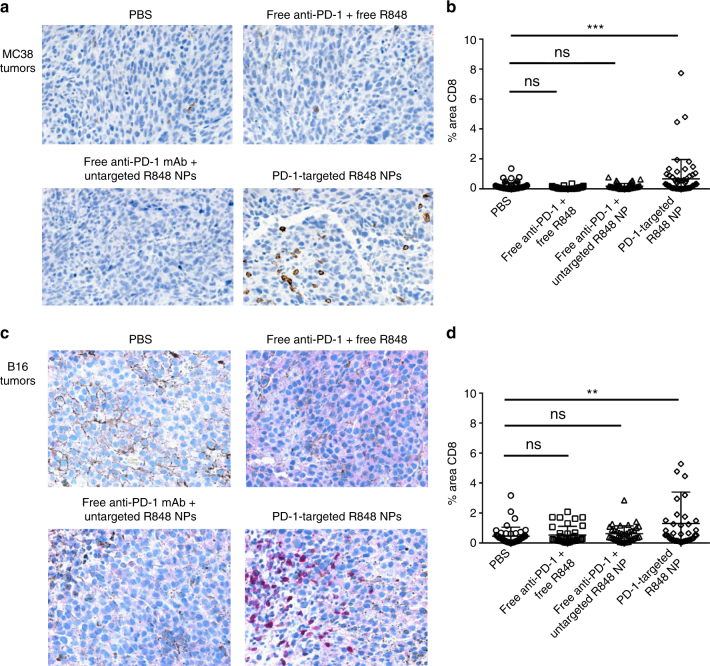
Targeted delivery of a TLR7/8 agonist (R848) to PD-1-expressing cells promotes infiltration of CD8^+^ T cells into MC38 and B16 tumors. C57BL/6 mice were inoculated subcutaneously with MC38 cells (**a**, **b**) or B16 cells (**c**, **d**). Fourteen days later, a single intravenous injection was performed with the following treatment groups: (1) PBS, (2) anti-PD-1 IgG and free R848, (3) anti-PD-1 IgG and untargeted particles loaded with R848, 4) PD-1-targeting particles loaded with R848. After 72 h, tumors were collected and processed into FFPE blocks for immunohistochemistry. **a**, **c** Immunohistochemistry using anti-CD8 reveals that tumors are not highly inflamed at baseline. An increase in TILs (quantified in **b**, **d** using ImageJ software, 10 random fields per mouse, 5 mice per treatment group) is observed only if the TLR7/8 agonist is delivered via the PD-1-targeting nanoparticles. The dose per injection was 20 μg of anti-PD-1 and 60 μg of R848, *n* = 50, mean ± s.d. (**p < 0.01; ***p < 0.001, one-way ANOVA with Tukey’s post hoc test)

The majority of cancer patients still do not respond to immunotherapy, and a major obstacle is the fact that many tumors are not inflamed by CD8^+^ T cells^30^. Delivery of inhibitors of immunosuppression—including inhibitors of TGFβ, IDO, and PD-L1—may have limited impact in the absence of tumor-infiltrating lymphocytes (TILs). We considered the possibility of inflaming a tumor microenvironment by leveraging the few PD-1^+^ cells that enter the tumors to deliver a Toll-like receptor (TLR) 7/8 agonist, R848 (resiquimod)^31^. After confirming the ability of the particles to sustain the release of R848 in vitro (Supplementary Fig. 8d), we transitioned to in vivo studies. Using the MC38 model, which has abundant CD3^+^ T cell infiltrates but very few CD8^+^ T cells in the cores of established (~400 mm^3^) tumors, we show that delivery of R848 loaded in PD-1-targeting nanoparticles results in an increase in CD8^+^ T cells, as determined by quantitative scoring of immunohistochemistry (Fig. 6a, b). Importantly, this approach was similarly effective in recruiting CD8^+^ T cells into completely non-inflamed established B16 tumors (Fig. 6c, d). Again, the effect was specific to targeted delivery of the payload to PD-1-expressing cells. Delivery of free antibody and free small molecule had no effect, nor did delivery of free anti-PD-1 in combination with R848 loaded in untargeted particles, suggesting that the nanoparticles do not passively accumulate in the tumors.

### Targeted delivery of R848 sensitizes the tumor to anti-PD-1

**Fig. 7:**
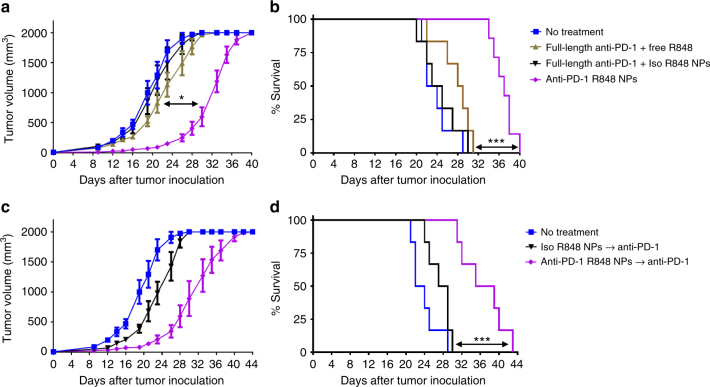
Targeted delivery of a TLR7/8 agonist (R848) to PD-1-expressing cells extends survival and sensitizes tumors to anti-PD-1. C57BL/6 mice were inoculated subcutaneously with 200,000 MC38 cells. **a**, **b** Starting on day 5, nanoparticles or free drugs were administered intravenously every other day up to a total of 10 injections. The dose was 20 μg of anti-PD-1 and 60 μg of R848 per injection. Tumor volume and animal survival were monitored to assess anti-tumor efficacy. **c**, **d** Nanoparticles or free drugs (20 μg of anti-PD-1 and 60 μg of R848) were administered intravenously on days 5, 7, and 9 post-tumor inoculation to activate the innate immune system and thereby inflame the tumor. On days 11, 14, and 17, anti-PD-1 antibody (clone 29 F.1A12, 200 μg) was injected intraperitoneally, as this IgG is typically administered. Tumor volume and animal survival were monitored to assess for efficacy (*n* = 6–7, mean ± s.e.m; *p < 0.05; ***p < 0.001, Mantel-Cox test)

Next, we sought to assess the therapeutic impact of targeted delivery of an agonist of innate immunity. Mice were inoculated with subcutaneous MC38 tumors and, beginning on day five, were administered anti-PD-1 and R848 intravenously at the modest doses of 20 μg anti-PD-1 and 60 μg R848, respectively, every other day for up to ten doses. Again, only delivery of R848 in PD-1-targeting nanoparticles delayed tumor growth (Fig. 7a) and extended mouse survival (Fig. 7b). We hypothesized that stimulating the innate immune system via targeted R848 delivery to the tumor microenvironment might increase sensitivity of the MC38 tumors to immune checkpoint blockade. Following three injections of R848-loaded nanoparticles that were either PD-1-targeting or untargeted on days 5, 7, and 9, free anti-PD-1 (200 μg/dose) was administered on days 11, 14, and 17. It was observed that targeted delivery of R848 primes the tumors to improves response to anti-PD-1 relative to untargeted delivery of R848 (Fig. 7c, d).

### Nanoparticles can be targeted to co-stimulatory receptors

Finally, we sought to demonstrate that additional cell lineages and functionalities could be targeted by these modular nanoparticles. We elected to target GITR, as this receptor allows us to assess whether the particles can agonize co-stimulatory receptors in addition to neutralizing co-inhibitory receptors such as PD-1. GITR is expressed predominantly by CD4^+^ Tregs^32^, and agonism of GITR has been shown to increase anti-tumor activity by decreasing the stability of the Treg lineage within tumors, thereby reducing immune suppression^33, 34^. We observed robust and specific binding of DiD-loaded, GITR-targeting nanoparticles to GITR-expressing CD4^+^ T cells recovered from B16 tumors 2 h after intravenous injection of the particles (Supplementary Fig. 10). These data confirm that this leukocyte-targeting platform is modular, as multiple receptors can be targeted and variable payloads can be delivered.

## Discussion

Unlike traditional approaches in oncology, immunotherapy can induce an adaptive response with capacity for memory. Adaptation is vital because cytotoxic agents select for resistant cancer clones, as tumors are heterogeneous and evolve when confronted with selection pressure^35^. Memory is crucial to achieving durable responses, preventing recurrence, which often leads to mortality. Cancer immunotherapy can generate a coordinated and proliferative anti-tumor response that has the potential to be applicable across varied cancer types and their underlying mutations^36^. Still, the fraction of patients who benefit from immunotherapy remains low, so new approaches that increase the therapeutic index are required.

Two main challenges that have limited the efficacy of immunotherapy to date are achieving therapeutically appropriate concentrations of drug among tumor-reactive lymphocytes and the lack of cytotoxic T lymphocytes in the tumors of many patients. To address the first obstacle, we used PD-1-targeting nanoparticles to focus the delivery of an inhibitor of immunosuppression whose target has pleiotropic effects. Loading the drug into targeted particles enables both the antibody and small molecule to be dosed at less than one-tenth of the standard dose, conferring efficacy and likely mitigating potential toxicity. To address the second obstacle, we used PD-1-targeting nanoparticles to deliver an agonist of innate immunity into the tumor microenvironment and thereby convert a “cold” tumor into a “hot” one.

The T cell-targeting nanoparticles described herein can concentrate immunomodulatory drugs at the site of immunosuppression following systemic administration. Whereas nanoparticles carrying cytotoxic payloads experience impaired diffusion into tumors^37^, T cells can penetrate deeply into the tumor parenchyma. Moreover, leukocytes are the first items that nanoparticles contact upon intravenous injection. As such, it is much more likely a targeting nanoparticle will bind to a receptor on an immune cell than to a receptor on a distant cancer cell that may be secluded behind dense extracellular matrix and high interstitial fluid pressure. Still, targeting of nanoparticles to P-selectin, which is expressed on stromal endothelial cells in addition to cancer cells, vastly improves the efficacy of cytotoxic agents relative to administration of free drug^38^, suggesting that targeting tumor vasculature may be a viable strategy as well.

Targeting of immune cells in vivo remains a nascent endeavor, particularly for delivery of small molecules. A previous study demonstrated that pre-incubation of LIF-containing particles targeted to CD4^+^ with splenocytes in vitro prior to adoptive cell transfer supported expansion of Foxp3^+^ Tregs as well as allograft survival^39^. Such nanoparticles could be administered intraperitoneally to increase the percentage of Tregs in lymphoid compartments^40^, though untargeted control particles were not included for comparison in either study. It is possible that Treg development could be induced by administration of free TGFβ and IL-2 or by sustained release of these two biologics from nanoparticles even in the absence of targeting to CD4^+^ cells. The data presented herein are the first to show targeted delivery of an immunomodulatory small molecule to endogenous immune cell subsets in vivo following intravenous administration, particularly to a dynamically expressed receptor such as PD-1, whose expression level is dictated by functional status.

While the proof-of-concept studies were conducted by targeting CD8 as a model receptor, therapeutics studies were performed by targeting PD-1. PD-1 is an attractive receptor for targeting, as PD-1 expression defines the tumor-reactive repertoire of T cells in tumors^19^ and in the circulation^20^. PD-1-targeting nanoparticles accumulate in tumors more effectively than isotype control particles (Fig. 3b), suggesting that the effect is at least partly mediated by the homing of PD-1^+^ T cells from the blood (Supplementary Fig. 6) into tumors.

Notably, the antibody fragments on the nanoparticles’ surface can be used not only to target specific T cell subsets but also to functionally neutralize co-inhibitory receptors. The particles can thus both induce immune checkpoint blockade and target the sustained release of complementary small molecules to inhibit other mediators of immunosuppression in an autocrine- and/or paracrine-like manner. The platform is modular, both in terms of payload and in terms of the targeting moiety. Co-stimulatory TNF receptor superfamily members—such as GITR, 4-1BB, OX40, and CD40—may be of particular interest, as their natural ligands are trimeric. While bivalent monoclonal antibodies (IgG) can agonize these receptors, the increased avidity afforded by the highly multivalent nanoparticles may enhance receptor crosslinking and hence the strength of stimulation of TNF receptor superfamily members^41^.

For the findings to be applied to the clinic, GMP-grade nanoparticles will have to be prepared, though relevant manufacturing procedures have already been established^7^. While the ability to target the delivery of multiple small molecules to T cells and/or the tumor microenvironment has been demonstrated through these studies, the selection of an appropriate small molecule payload will be crucial to maximizing the benefit afforded by this platform. While TGFβ is often overexpressed in colorectal tumors^42^, inhibition of TGFβR1 did not lead to curative outcomes under the conditions examined. It has been suggested that elevated levels of TGFβ may foster the development of excessive stromal features that can prevent the penetration of leukocytes into the tumor parenchyma^43^. The immunohistochemistry data revealed that there are very few CD8^+^ T cells in established MC38 tumors, suggesting that more favorable survival outcomes might be achieved in a tumor with more CD8^+^ TILs or upon administration of a different payload. For example, one might consider the targeted delivery of an epigenetic modifying drug that can reverse the exhaustion of PD-1^hi^CD8^+^ T cells and improve T cell responses and tumor control upon immune checkpoint blockade^44^. The full synergistic potential of such combination therapies may be realized by optimizing the formulation with regards to the choice and dose of the small molecule and the functional antibody. A comprehensive formulation campaign will enable the determination of defined concentrations and release profiles of encapsulated drugs, surface density of the targeting antibody fragment, and size and charge of the nanoparticles.

In summary, a robust in vivo T cell-targeting drug delivery system has been developed. Specific and efficient binding is observed in vitro, including to human cells, and in vivo. Such binding allows for targeted delivery of an inhibitor of TGFβR1 or an agonist of TLR7/8, delaying tumor growth and extending survival of tumor-bearing mice if and only if the small molecule is delivered via PD-1-targeting nanoparticles, as free compounds and untargeted particles had no effect. Excitingly, this platform can be used to deliver payloads *in trans* to the tumor microenvironment in a paracrine-liker manner. Targeted delivery of a R848, which is less hydrophobic than SD-208 and is thus released prior to any meaningful amount of nanoparticle uptake, promotes infiltration of CD8^+^ T cells into MC38 and B16 tumors. T cells do not express TLR7/8, so the observed recruitment of CD8^+^ T cells was likely owing to local activation of plasmacytoid dendritic cells, which are known to secrete IFNα and IL-12 in response to treatment with R848^45^. R848 also induces differentiation of myeloid-derived suppressor cells into macrophages and dendritic cells^46^, which may account for the improved efficacy of subsequent anti-PD-1 therapy. Together, these data suggest that targeted delivery of immunotherapy to endogenous immune cell subsets can improve therapeutic index and may be worthy of additional investigation, particularly with regards to breaking immune tolerance and increasing the proportion of patients who respond to cancer immunotherapy.

## Methods

### Nanoparticle formulation

PLGA-based nanoparticles were prepared using single-emulsion evaporation. PLGA (AP041, acid end-capped, 50:50, 10–15 kDa, Akina) was blended with Mal-PEG-PLGA (AI53, diblock copolymer, 50:50, 5–10 kDa, Akina) at 25% w/w. The polymers were dissolved in 1 mL dichloromethane (Sigma) and added to 6 mL of ice-cold 0.25% PVA (30,000–70,000 g mol^−1^, Sigma) in 50 mM phosphate buffer, pH 5.8. The two phases were emulsified using a sonic probe (Qsonica Q700 with microtip, amplitude 10, 3 s power with 2 s break). SD-208- and R848-loaded nanoparticles were prepared by adding 10% (w/w) SD-208 (Selleckchem) or 40% (w/w) R848 (Sigma) to the solvent/polymer phase. The emulsion was stirred at room temperature for 3 h to evaporate the dichloromethane and afterwards purified by two wash-spin cycles in PBS at 20,000 *g* for 10 min. Nanoparticles were assessed for size distribution and zeta potential using a Zetasizer Nano series ZS90, and drug encapsulation was determined by absorbance at 370 nm (SD-208) or 280 nm (R848). The morphology of freeze-dried samples was assessed by Scanning Electron Microscopy (SEM; JSM-7800F Prime, Jeol, Japan).

### Antibody cleavage and conjugation

IdeS and IdeZ (obtained from Genovis or Promega) were used for site-specific cleavage of full-length IgG antibodies into F(ab’)2 and Fc. IdeS was used for the anti-CD8a (BioXCell, YTS169.4), rat IgG2b isotype control (BioXCell, LTF-2), pembrolizumab (DFCI), human IgG4 isotype control (BioLegend, ET904), anti-GITR (BioLegend, DTA-1), and anti-Gr-1 (BioLegend, RB6-8C5). IdeZ was used for anti-PD-1 clone 332.6D2 from Dr. Gordon Freeman (DFCI) and mouse IgG2a isotype control (BioXCell, C1.18.4). Antibodies were diluted in PBS with 5 mM EDTA to 1–4 mg mL^−1^ and incubated for 1–2 h at the recommended concentration of 1 unit enzyme per µg of antibody at 37 °C. Antibody cleavage was confirmed by non-reducing SDS–PAGE. The antibody fragments were then reduced using 0.5 mM dithiothreitol (DTT, Sigma) for 30 min at 25 °C to retrieve free sulfhydryl groups for chemical linkage to the maleimide group on the nanoparticle surface. Free DTT was removed before conjugation using 7 kDa desalting columns (Thermo Scientific). Antibody concentration was measured by NanoDrop (Thermo Scientific), and 25 µg of antibody was added per 1 mg of polymer. The reaction was carried out for 2 h at 25 °C under shaking. The amount of antibody on the nanoparticle surface was quantified by BCA assay (Thermo Scientific). Western blot (following reducing SDS–PAGE) was performed to confirm the absence of Fc on the nanoparticle surface using Fab- or Fc-specific antibodies (Jackson ImmunoResearch, 112-035-008 and 112-035-006; diluted 1:20,000 for the detection of rat-derived antibodies and 1:5,000 for the mouse-derived antibodies). All cleavage reactions and following analysis using gels and Western blots were performed independently at least twice.

### Cell culture

Murine T cells were enriched from spleens using the EasySep CD8 T cell enrichment kit (StemCell Technologies) and cultured in RPMI-1640 media supplemented with 10% FBS, 1% penicillin–streptomycin, 1% GlutaMAX, 10 mM HEPES, 1 mM sodium pyruvate, and 55 μM 2-mercaptoethanol. B16-F10 (ATCC) and MC38 (obtained from Dr. Wucherpfennig’s lab) were cultured in DMEM supplemented with 10% FBS and 1% penicillin-streptomycin. The media for ovalbumin-expressing B16 cells (obtained from Dr. Wucherpfennig’s lab) was further supplemented with 0.5 mg mL^−1^ geneticin. All cell lines were tested for mycoplasma and confirmed to be negative. All supplements were obtained from Life Technologies. Blood collars for human T cells were obtained from the Brigham and Women’s Hospital Blood Donor Center. Appropriate consent was obtained from all donors. T cells were enriched using the Rosette Sep Human T cell enrichment kit, and cells were separated via ficoll gradient separation using SepMate. T cells were cultured in ImmunoCult-XF T Cell Expansion Medium supplemented with 10 ng mL^−1^ IL-2 (Peprotech) and activated with 25 µl mL^−1^ ImmunoCult Human T Cell Activator (all from StemCell Technologies). The purity of the isolated cells was determined using anti-human CD3 antibody (BioLegend) and confirmed to be greater than 95% purity.

### In vitro T cell assays

In vitro binding of nanoparticles was assessed after incubation of 250,000 enriched CD8 T cells with fluorescently (DiD, Life Technologies) labeled nanoparticles at different concentrations for 30 min at 37 °C. After the incubation, T cells were washed 3 to 5 times in PBS and directly assessed by flow cytometry for DiD fluorescence. For internalization studies, cells were incubated for 30 min on ice, washed, and either stained immediately with AF488-anti-F(ab’)2 antibody or incubated for the indicated periods of time at 37 °C before secondary staining. AF488-anti-F(ab’)2 secondary antibodies were purchased from Jackson ImmunoResearch Laboratory (product 109-545-097 for anti-human PD-1, 112-545-006 for anti-rat CD8a; both diluted 1:100). T cells isolated from OT-I Rag1^−/−^ mice were activated by Dynabeads Mouse T-Activator CD3/CD28 (Thermo Scientific) at a ratio of 2:1 T cell to bead or by ova-expressing B16 melanoma cells at a ratio of 10:1 T cell to B16 cell. Carboxyfluorescein succinimidyl ester (CFSE, BioLegend) or Cell Trace Violet (Thermo Scientific) was used to assess T cell proliferation; the labeling was carried out according to the manufacturer’s recommendations. Mouse TGFβ1 was purchased from Cell Signaling Technologies, and T cell supernatants were analyzed by mouse IFNγ ELISA MAX™ (BioLegend).

### Flow cytometry

The following antibody clones were used for assessments by flow cytometry (BD LSR Fortessa) using murine T cells: mCD8a 53–6.7, mCD8b YTS156.7.7, mCD4 GK1.5, mCD3e 145-2C11, mCD3 17A2, mPD-1 29 F.1A12, mGranzyme B GB11, mCD45 30-F11, mCD62L MEL-14, mCD44 IM7, mGITR YGITR.765, mCD11b M1/70, mF4/80 T45-2342, mCD3e 17A2, mNK1.1 PK136, mIFNγ XMG1.2, and mZAP-70 1E7.2. The following clones were used for experiments involving human T cells: hPD1 EH12.2H7, hIFNy B27, hCD3 HIT3a. Zombie Aqua™ (diluted 1:200) was used as a live/dead stain. With the exception of clones T45-2342 and 17A2 (BD Biosciences), all antibodies were purchased from BioLegend. Antibodies were diluted 1:100 for surface staining and 1:50 for intracellular staining.

### Animal experiments

All animal experiments were carried out according to protocols approved by Dana-Farber Cancer Institute, Institutional Animal Care and Use Committee (IACUC). Six-to-ten week-old C57BL/6 mice were purchased from Jackson Laboratory. OT-I Rag1^−/−^ were obtained from in-house breeding. Both male and female mice were used for the studies. For experiments designed to assess nanoparticle binding, 400,000 B16 melanoma cells were inoculated subcutaneously into the flanks of the mice. When the tumors had grown to ~400 mm^3^ (tumor volume calculated as ½ × length × width^2^), nanoparticles were administered intravenously. One, two, 24, or 48 h later, tumors were cut into small pieces, and extracellular components were digested by addition of collagenase type IV (~50 units per mL, Thermo Scientific) and DNase (~20 units per mL, Roche). Tumor samples were homogenized using gentleMACS for 37 s. Cell suspensions of spleens and TdLNs were generated using a 70 μm strainer, and blood was collected via cardiac puncture. Red blood cells were removed by ACK buffer (Life Technologies) for all mouse tissue samples. For experiments designed to assess therapeutic efficacy, 200,000 MC38 cells were inoculated subcutaneously into the flanks of the mice. After 5 days, nanoparticles or free drugs were administered intravenously every other day up to a total of 10 injections. 2 mg of nanoparticles were administered, translating to a dose of 20 μg anti-PD-1 and 40 μg SD-208. At least eight mice were included in each group. For experiments designed to assess the ability to “warm” a tumor microenvironment, 200,000 MC38 cells or 200,000 B16 cells were inoculated subcutaneously into the flanks of the mice. After 14 days, nanoparticles or free drugs were administered intravenously, and tumors were recovered 72 h later. 2 mg of nanoparticles were administered, translating to a dose of 20 μg anti-PD-1 and 60 μg R848. For the second in vivo study with R848, in which targeted particles were used to sensitize the tumors to immune checkpoint blockade, the nanoparticles were administered intravenously on days 5, 7, and 9 and the anti-PD-1 antibody clone 29 F.1A12 was administered intraperitoneally at 200 μg per dose on days 11, 14, and 17. Therapeutic studies were performed at least in duplicate and included at least six mice per group. Animals were randomized into cages of five prior the first injection, and mice were excluded from the study if they had no measurable tumor on day 5 post inoculation. No treated animals or samples were excluded from the analysis. The injections were performed by a blinded investigator. Every data point represents data obtained from one mouse.

### Immunohistochemistry

Tumors were fixed in a 10% formalin solution (Sigma) for 24 h and then transferred into 70% ethanol. Samples were cut into 5 μm-thick sections, and antigen retrieval was performed on a Leica automated immunohistochemistry staining platform using PH6 Citrate. Slides were stained using anti-CD8 (eBioscience, clone 4SM15) at 1:100 dilution on a Leica Bond automated immunohistochemistry staining platform, followed by staining with DAB (Millipore, rat HRP, 1:100) for MC38 tumors or with Code ×0909 protein block (DAKO, rabbit, 1:750) for B16 tumors. For the latter, staining of red chromagen was achieved using the Leica Bond Polymer Refine Red detection kit. Mouse spleen was used as control tissue. Slides were imaged on an inverted fluorescence microscope using the AxioVision microscopy software (Zeiss), and quantification was performed by analyzing 10 randomly selected images per slide using ImageJ.

### Statistical analysis

A two-tailed Student’s *t*-test or one-way ANOVA with Tukey’s post hoc test was used to determine statistical significance between two groups and several groups, respectively. For survival studies, the log-rank (Mantel-Cox) test was used to compare drug-loaded anti-PD-1 nanoparticles with no treatment. Analyses were performed using GraphPad Prism 7.01.

### Data availability

The authors declare that the data supporting the findings of this study are available within the article and its Supplementary Information Files or from the corresponding author on reasonable request.

## Electronic supplementary material

Supplementary Information
