# Supplementary material for: T cell-targeting nanoparticles focus delivery of immunotherapy to improve antitumor immunity
Source: Nat Commun. 2017 Nov 23;8:1747. doi: 10.1038/s41467-017-01830-8 (PMC5700944; doi:10.1038/s41467-017-01830-8)
Supplement: Supplementary file 1 — Supplementary Information [file 41467_2017_1830_MOESM1_ESM.pdf]

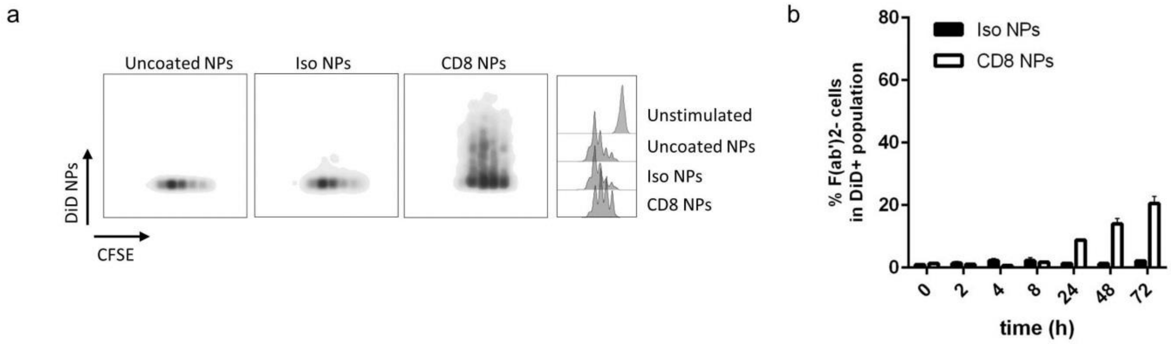

Supplementary Figure 1. T cells retain their ability to proliferate in co-culture with ovalbumin-expressing B16 melanoma cells and internalize CD8a-targeting nanoparticles. (**a**) OT-I CD8+ T cells were incubated with anti-CD8a nanoparticles (or relevant negative controls) for 30 min, washed to remove unbound nanoparticles, and co-cultured with ovalbumin-expressing B16 tumor cells for 72 h. Proliferation was assessed by CFSE dilution, and nanoparticle binding was assessed by fluorescence of DiD, which had been entrapped in the nanoparticle core. (**b**) Percentage of F(ab')2-negative cells in the DiD-positive population to assess internalization of anti-CD8a nanoparticles, n=3 for 72 h, n=4 for 0-48 h, mean ± s.e.m.

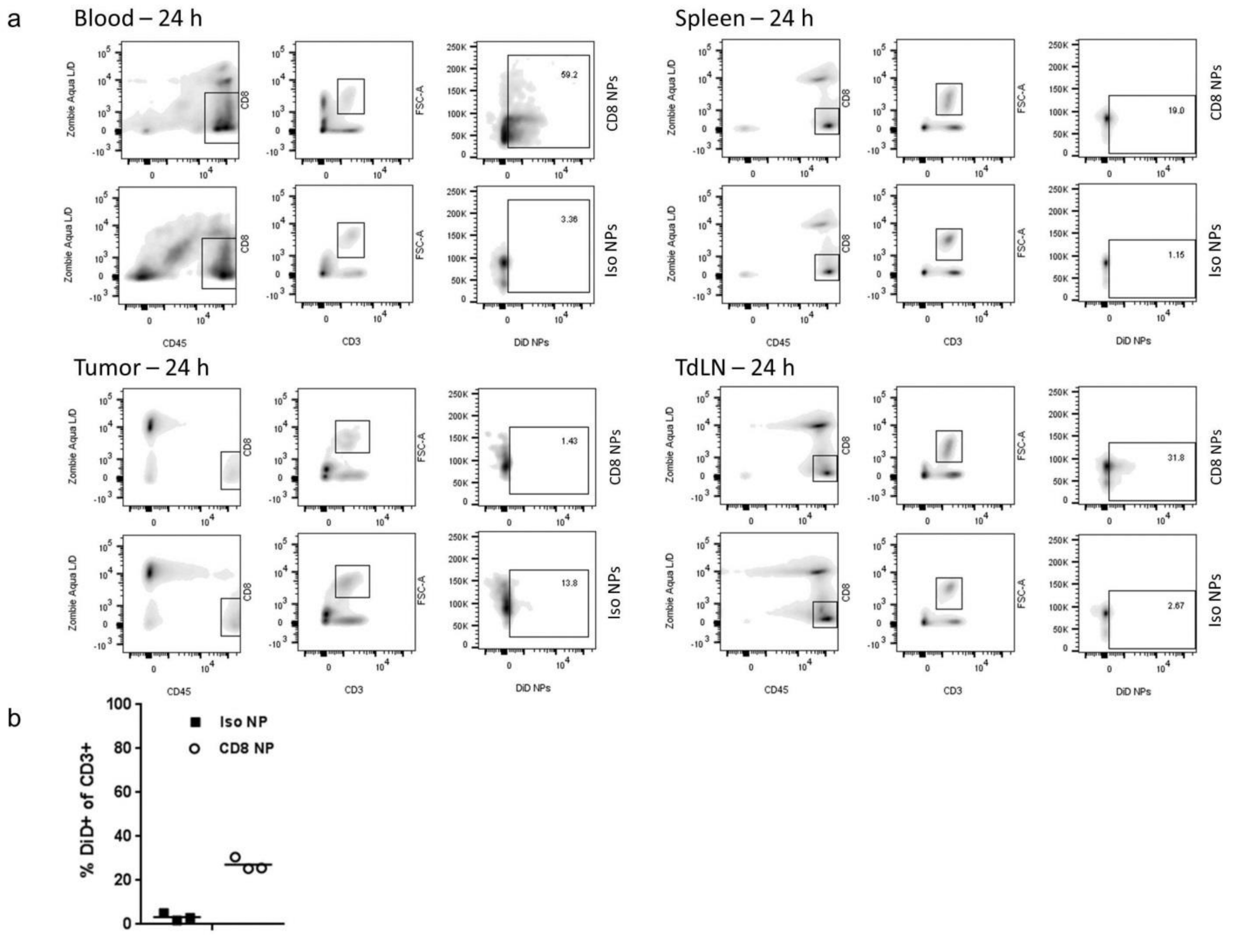

Supplementary Figure 2. *In vivo* assessment of anti-CD8a nanoparticles among T cells.
(**a**) Gating strategy of *in vivo* binding experiment for blood, spleen, tumor, and TdLN. (**b**) Percentage of nanoparticle-bound CD3$^+$ T cells in the blood after nanoparticles were in the circulation for 1 h, as described in Figure 2d.

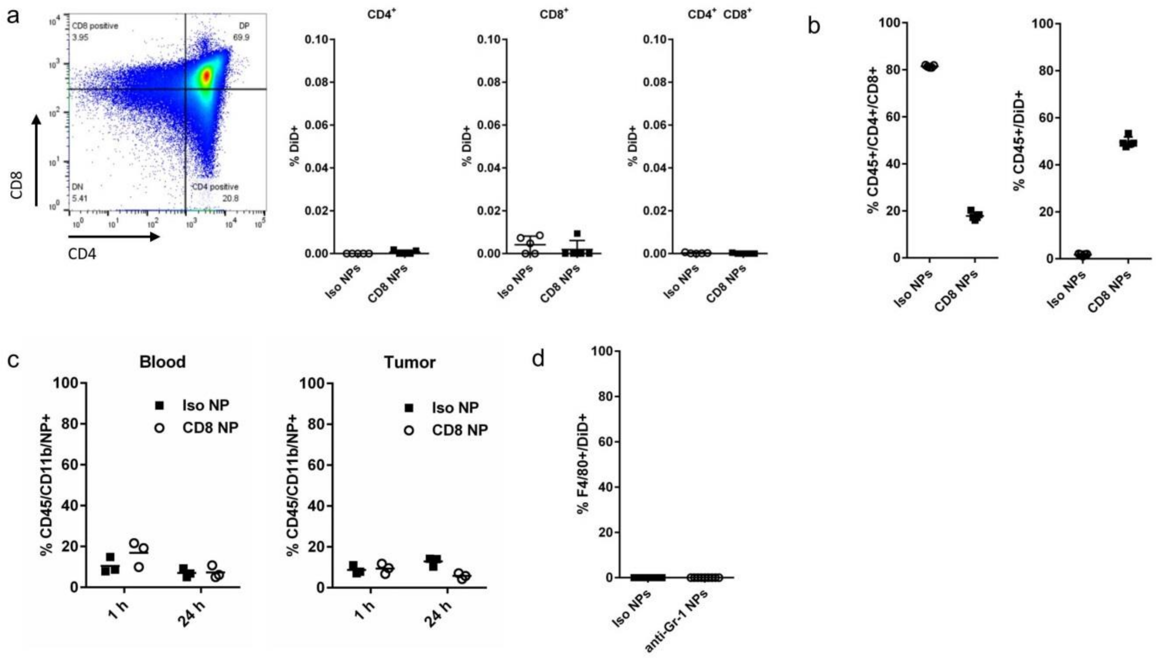

Supplementary Figure 3. *In vivo* distribution of anti-CD8a nanoparticles to thymus and myeloid cells. (**a**) Anti-CD8a nanoparticles were injected intravenously, and cells from the thymus were analyzed for DiD fluorescence after 1 h (n=5, mean ± s.d.). (**b**) Mouse thymocytes were isolated and incubated *ex vivo* with DiD-loaded anti-CD8a or isotype control nanoparticles. Double-positive T cells could only be stained for CD8 in the absence of anti-CD8a nanoparticles due to steric hindrance, as observed in Figure 2d (left panel). Gated on $CD45^+$ cells, there is significant higher number of DiD^+ cells for anti-CD8 nanoparticles (right panel). (**c**) Anti-CD8a and isotype nanoparticles were injected intravenously, and $CD11b^+$ cells were analyzed for DiD fluorescence 1 h and 24 h after injection. (**d**) $CD11b^+$ myeloid cells gated on $F4/80^+$ and DiD^+ are shown. In the absence of Fc (IgG constant region), the anti-GITR F(ab')2 nanoparticles are not recognized by Fc receptors expressed on macrophages, as neither phagocytosis nor non-specific binding is observed.

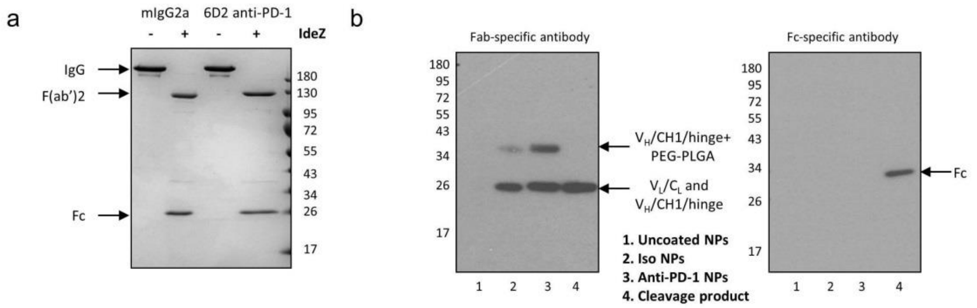

Supplementary Figure 4. Characterization of PD-1-targeting nanoparticles. (**a**) A non-reducing SDS-PAGE gel stained with Coomassie Brilliant Blue following enzymatic cleavage of anti-PD-1 and mouse IgG2a antibodies using IdeZ. (**b**) A Western blot after reducing SDS-PAGE of PD-1-targeting nanoparticles developed with Fab-specific (left panel) or Fc-specific antibodies (right panel); lane 1: uncoated nanoparticles, lane 2: isotype nanoparticles, lane 3: anti-PD-1 nanoparticles, lane 4: anti-PD-1 F(ab')2 and Fc cleavage product as positive control.

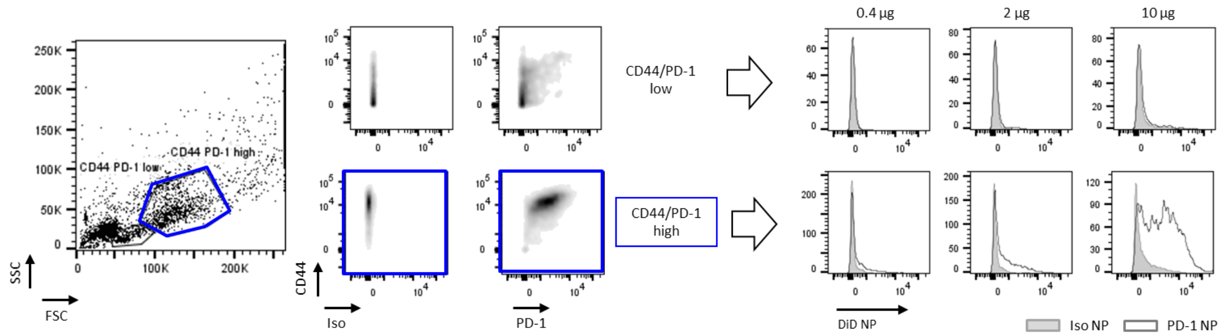

Supplementary Figure 5. Binding of PD-1-targeting nanoparticles to T cells activated by anti-CD3/CD28 beads. $CD8^+$ OT-I T cells were activated with CD3/CD28 beads (ratio 1:2 beads to T cell) for 48 h and incubated with DiD-loaded, PD-1-targeting nanoparticles for 30 min before detection of DiD by flow cytometry. Mass of polymer indicated is per 250,000 T cells.

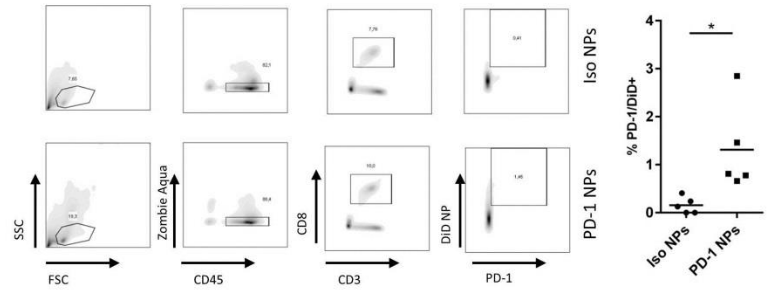

Supplementary Figure 6. Binding of PD-1-targeting nanoparticles to T cells in the circulation of B16 tumor-bearing mice. C57BL/6 mice were inoculated with ovalbumin-expressing B16 melanoma cells. Once tumors reached ~400 mm^3 in volume, DiD-loaded, PD-1-targeting nanoparticles were injected intravenously. One hour later, blood was recovered. Flow cytometry was performed (gating shown at left), and the percentage of T cells that were positive for both PD-1 expression and nanoparticle binding was quantified (right panel) (* $p < 0.05$, Two-tailed student's t-test).

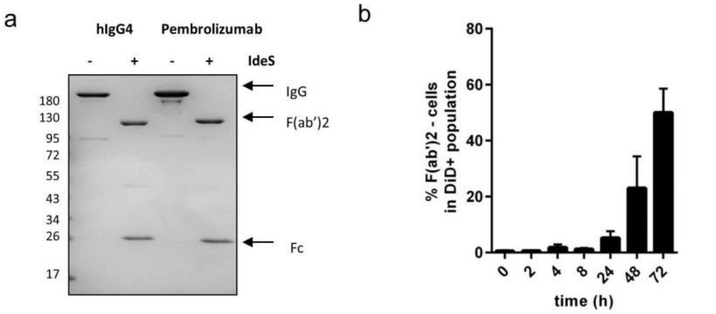

Supplementary Figure 7. Generation and characterization of anti-human PD-1 nanoparticles. (**a**) A non-reducing SDS-PAGE gel stained with Coomassie Brilliant Blue following enzymatic cleavage of pembrolizumab and human IgG4 antibodies using IdeS. (**b**) Percentage of F(ab')2-negative cells in the DiD-positive population to assess internalization of PD-1-targeting nanoparticles, n=2 for 72 h, n=3 for 48 h and n=4 for 0-24 h, mean ± s.e.m.

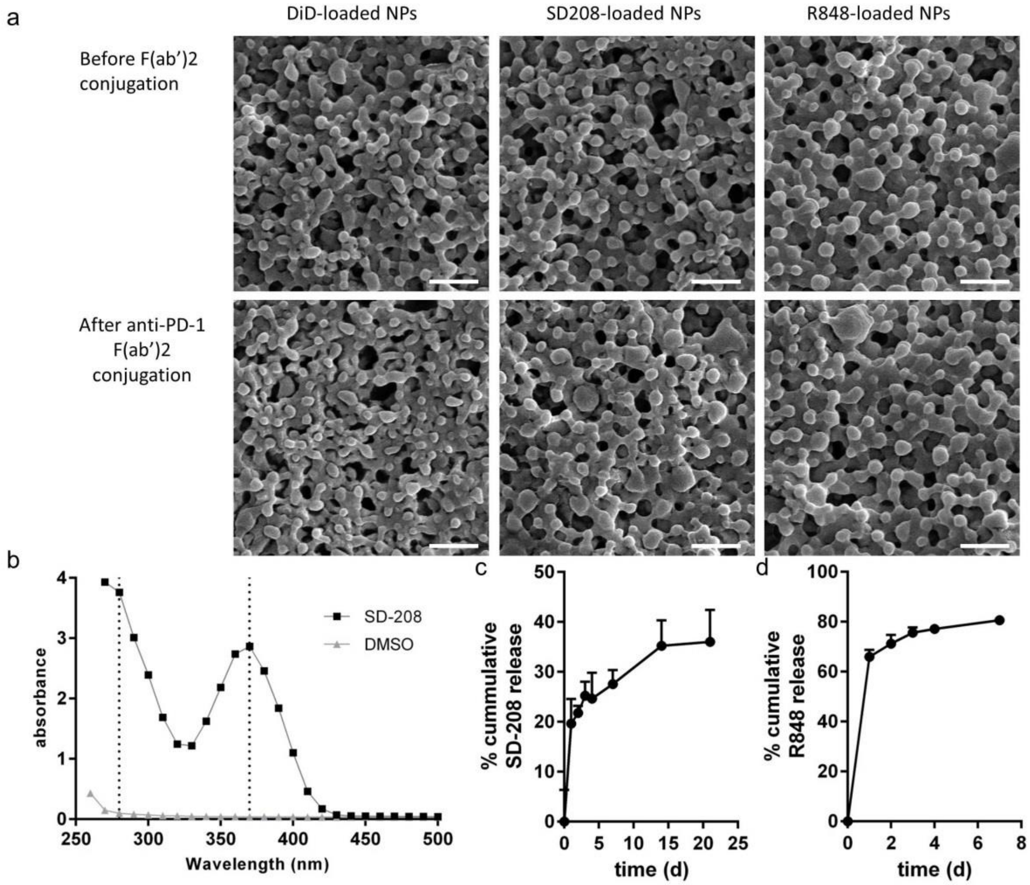

Supplementary Figure 8. Analysis of small molecule-encapsulating nanoparticles.
(**a**) Scanning electron microscopy images of nanoparticle formulations loaded with DiD, SD-208, or R848 before and after F(ab')2 conjugation; scale bar = 500 nm. (**b**) Absorbance scan of SD-208 for the determination of drug encapsulation. (**c**) Release profile of SD-208-loaded nanoparticles in 10% FBS in PBS, confirming the particles' ability to sustain release of the entrapped payload, n=3, mean ± s.d. (**d**) Release profile of R848-loaded nanoparticles in 10% FBS in PBS, n=3, mean ± s.d.

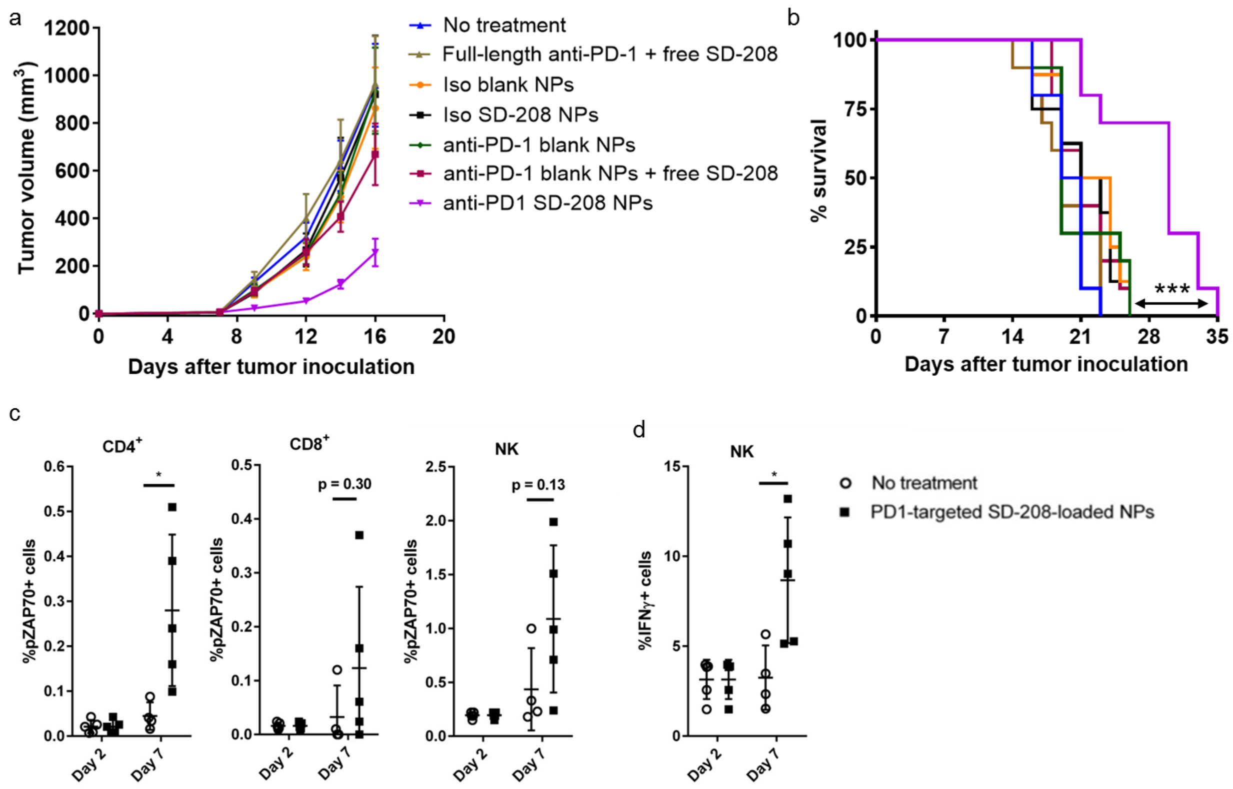

Supplementary Figure 9. Targeted delivery of a TGFβR1 inhibitor (SD-208) to PD-1-expressing cells delays tumor growth and extends survival even with decreased dosing frequency. C57BL/6 mice were inoculated subcutaneously with 200,000 MC38 cells. Five days later, nanoparticles or free drugs were administered intravenously twice weekly up to a total of 7 injections. The dose was 20 µg of anti-PD-1 and 40 µg of SD-208. (**a**) Tumor volume and (**b**) animal survival were monitored to assess efficacy (n=8-10, mean ± s.e.m.; *** $p < 0.001$, Mantel Cox test). (**c, d**) On day 2 (before treatment) and on day 7 (two days after the first treatment), blood was collected. $CD4^+$ T cells, $CD8^+$ T cells, and NK cells were analyzed for pharmacodynamic markers of PD-1 blockade (n=5, mean ± s.e.m.; * $p < 0.05$, Two-tailed student's t-test).

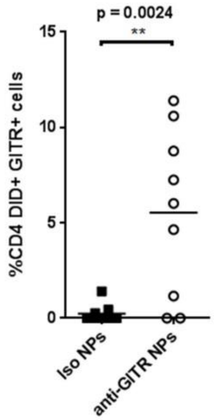

Supplementary Figure 10. Nanoparticles can be targeted to $GITR^+$ $CD4^+$ T cells. C57BL/6 mice were inoculated subcutaneously with B16 melanoma cells. Once tumors reached ~400mm^3 in volume, DiD-loaded, GITR-targeting nanoparticles were injected intravenously. Two hours later, tumors were recovered. Flow cytometry was performed, and the percentage of $CD4^+$ T cells that were positive for both target (GITR) expression and nanoparticle binding (DiD) was quantified ($n = 9$; ** $p < 0.01$, Two-tailed student's t-test).

Supplementary Table 1. Zeta potential of nanoparticle formulations before and after F(ab')2 conjugation, measured in H_2O.

	Before conjugation		After conjugation	
	average (mV)	s.d. (mV)	average (mV)	s.d. (mV)
**Blank NP**	-1.910	0.259	-4.725	0.406
**DiD**	-7.013	0.457	-7.692	0.371
**SD208**	-2.037	0.403	-3.725	0.274
**R848**	-10.303	0.306	-12.450	0.310
**DiR**	-7.755	0.260	-5.495	0.439
